# Platinum single-atom adsorption on graphene: a density functional theory study†

**DOI:** 10.1039/c8na00236c

**Published:** 2019-01-08

**Authors:** Sasfan Arman Wella, Yuji Hamamoto, Suprijadi, Yoshitada Morikawa, Ikutaro Hamada

**Affiliations:** Department of Precision Science and Technology, Graduate School of Engineering, Osaka University 2-1 Yamada-oka, Suita Osaka 565-0871 Japan ihamada@prec.eng.osaka-u.ac.jp; Department of Physics, Faculty of Mathematics and Natural Sciences, Institut Teknologi Bandung Jalan Ganesha 10 Bandung 40132 Indonesia; Element Strategy Initiative for Catalyst and Batteries, Kyoto University Katsura Kyoto 615-8520 Japan; Research Center for Ultra-Precision Science and Technology, Graduate School of Engineering, Osaka University 2-1 Yamada-oka, Suita Osaka 565-0871 Japan

## Abstract

Single-atom catalysis, which utilizes single atoms as active sites, is one of the most promising ways to enhance the catalytic activity and to reduce the amount of precious metals used. Platinum atoms deposited on graphene are reported to show enhanced catalytic activity for some chemical reactions, *e.g.* methanol oxidation in direct methanol fuel cells. However, the precise atomic structure, the key to understand the origin of the improved catalytic activity, is yet to be clarified. Here, we present a computational study to investigate the structure of platinum adsorbed on graphene with special emphasis on the edges of graphene nanoribbons. By means of density functional theory based thermodynamics, we find that single platinum atoms preferentially adsorb on the substitutional carbon sites at the hydrogen terminated graphene edge. The structures are further corroborated by the core level shift calculations. Large positive core level shifts indicate the strong interaction between single Pt atoms and graphene. The atomistic insight obtained in this study will be a basis for further investigation of the activity of single-atom catalysts based on platinum and graphene related materials.

## Abstract 2

Single-atom catalysis, which utilizes single atoms as active sites, is one of promising ways to enhance the catalytic activity and to reduce the amount of precious metals used. Here by means of density functional theory based thermodynamics we show that the single platinum atoms preferentially adsorb on the substitutional carbon sites at the hydrogen terminated graphene edge.

## Graphical abstract

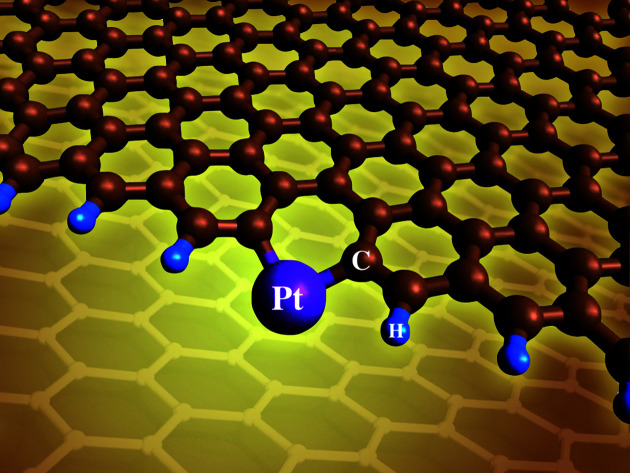

## 1 Introduction

High-performance electrocatalysts are highly desired for electrochemical energy conversion devices, such as photovoltaic cells and fuel cells. Platinum (Pt) is widely used as an electrocatalyst, as it exhibits high catalytic activity not only for hydrogen oxidation but also for oxygen reduction at low temperatures.^1,2^ Nevertheless, there is still an urgent need to address the high cost of Pt and to search for alternative catalysts, which use small amounts of Pt or no Pt with earth-abundant materials. Tremendous efforts have been devoted to achieve these goals, including the use of non-precious metals for Pt–metal alloys,^3–9^ or the use of noble metal-free catalysts.^10–13^ Particularly interesting are Pt clusters supported by graphitic materials, such as carbon black, carbon nanotubes, and graphene, which have been extensively studied both experimentally,^14–20^ and theoretically.^21–34^ A better catalytic activity of small Pt clusters supported by graphene sheets has been demonstrated experimentally.^16,17^ These results lead to the hypothesis that downsizing the Pt clusters to single atoms can enhance the catalytic activity. Recently, Sun *et al.* have successfully deposited single Pt atoms on graphene nanosheets using atomic layer deposition and then demonstrated a significant improvement of the catalytic activity for the methanol oxidation reaction.^35^ Cheng *et al.* also showed that a Pt single-atom catalyst deposited on nitrogen doped graphene nanosheets exhibits enhanced catalytic activity for the hydrogen evolution reaction.^36^ The vacancies usually formed during the preparation are expected to have strong interactions with the Pt atoms as demonstrated in several theoretical studies.^23,31,32^ Back *et al.* have predicted the great potential of a single atom catalyst supported on defective graphene for CO_2_ electroreduction applications.^37^ However, the dispersion of the single Pt atoms on graphene is limited to the number of point defects. On the other hand, the graphene edge might offer more space for depositing single Pt atoms. Kong *et al.* have theoretically investigated Pt single-atom adsorption at the edges of graphite nanofibers and found that the atoms are tightly bound to the edges due to the existence of active dangling bonds.^38^ By employing transmission electron microscopy, the structure and dynamics of Au,^39^ Fe,^40^ Cu,^41^ and Pt^41,42^ atoms at the edges have been studied. Some experimental studies also observed that Pt nano-clusters at the graphene edges are stable at high-temperatures.^43–45^ However, the adsorption state and the catalytic activities of single Pt atoms are not yet fully understood.

In this work, we investigate Pt single-atom adsorption on graphene by means of density functional theory (DFT) that includes van der Waals forces. We perform systematic calculations to determine the adsorption state of single Pt atoms on graphene, including defective graphene structures and graphene edges. Special emphasis is devoted to the graphene edges, as they are abundant under realistic conditions. We consider both zigzag and armchair edges, including the dependence on hydrogen termination. We examine their stability by taking into account the environmental effects, *via* DFT based thermodynamics.^46^ We find that single Pt atoms adsorb preferentially at the edge rather than on graphene, and the substitutional carbon site is the most stable one under the conditions relevant to experiments. Furthermore, the core level shift (CLS) of Pt atoms is calculated for each structure, which is used to validate the predicted structure against the experiment.

## 2 Computational details

All the DFT calculations are performed by using STATE code^47,48^ with ultrasoft pseudopotentials.^49^ A plane-wave basis set is used to expand wave functions and charge density with cutoff energies of 36 Ry and 400 Ry, respectively. We use rev-vdW-DF2 (ref. 50) exchange correlation functional as implemented^51^ in the code with an efficient algorithm.^52,53^ Pseudopotentials are generated using the Perdew–Burke–Ernzerhof (PBE)^54^ functional and the use of PBE pseudopotentials in rev-vdW-DF2 calculations is validated in ref. 55. We use a (6 × 6) supercell to simulate Pt single-atom adsorption on pristine (GR), mono-carbon vacancy (*V*_1_), and di-carbon vacancy (*V*_2_) graphene structures. We introduce the 7575 membered rings of graphene as a boundary between zigzag and armchair as observed in ref. 56, denoted as grain-boundary graphene (GB-GR). We place the GB-GR in a 17.23 Å × 14.03 Å rectangular unit cell. We also construct disorder graphene (DisGR) reported in ref. 57, and place it in a 20.37 Å × 24.67 Å rectangular cell. Brillouin zone integration is performed for all structures using the *Γ*-centered 4 × 4 *k*-point mesh for pristine, mono-carbon vacancy, and di-carbon vacancy graphenes; 2 × 3 *k*-point mesh for GB-GR; and the *Γ*-point for DisGR. To investigate the edge effect in the Pt adsorption, we employ graphene nanoribbons (GNRs) with zigzag (*z*GNR) and armchair (*a*GNR) edges with different terminations, including non-hydrogenated, mono-hydrogenated, and di-hydrogenated ones. We follow the convention used in ref. 58, *i.e.*, *z*_*n*_ to denote *z*GNR with *n* hydrogen atoms at the edge carbon (C) site, and *a*_*n*_ for *a*GNR with *n* hydrogen (H) atoms. Spin polarization is taken into account for all systems. *z*GNR has the localized spin with ferromagnetic order along the edge and antiparallel orientation between the edges as reported in ref. 59 and 60, while such localized spin does not appear at the edge of *a*GNR. GNRs are modeled using a periodic supercell along the edges, having the same configurations on both edges. Following the conventional notation, the width of GNR is specified by the number of zigzag chains and dimer lines for *z*GNR and *a*GNR, respectively, and we use 5-*z*GNR and 10-*a*GNR in this work. We use supercells containing 4 hexagons for both *z*GNR and *a*GNR, and the resulting supercells contain 40 carbon atoms. Supercells in the graphene plane directions correspond to 
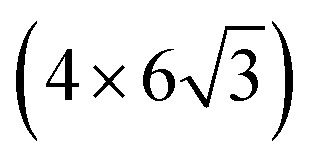
 and 
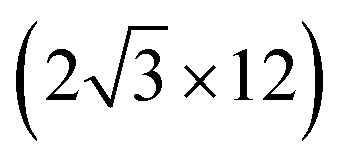
 supercells of graphene for *z*GNR and *a*GNR, respectively, and graphene planes are separated by a vacuum of ∼15 Å thickness. Vacuum thicknesses between edges are 16.48 Å and 18.70 Å for *z*GNR and *a*GNR, respectively. Brillouin zone integration is performed using the *Γ*-centered 6 × 2 *k*-point set for both *z*GNR and *a*GNR. All the graphene based structures considered in this work are constructed using the lattice constant of graphene obtained using rev-vdW-DF2 (2.46 Å),^61^ which is in good agreement with the experimental value for graphite (2.4589 ± 0.0005 Å).^62^ The structures are fully relaxed until the forces acting on the atoms become smaller than 5.14 × 10^−2^ eV Å^−1^ (1 × 10^−3^ Hartree/Bohr). The Pt 4f CLS including the final state screening is calculated as the difference between the core level binding energy of Pt adsorbed GNR and bulk Pt.^63,64^

## 3 Results and discussion

### 3.1 Pt single-atom adsorption on pristine and defective graphene structures

**Fig. 1:**
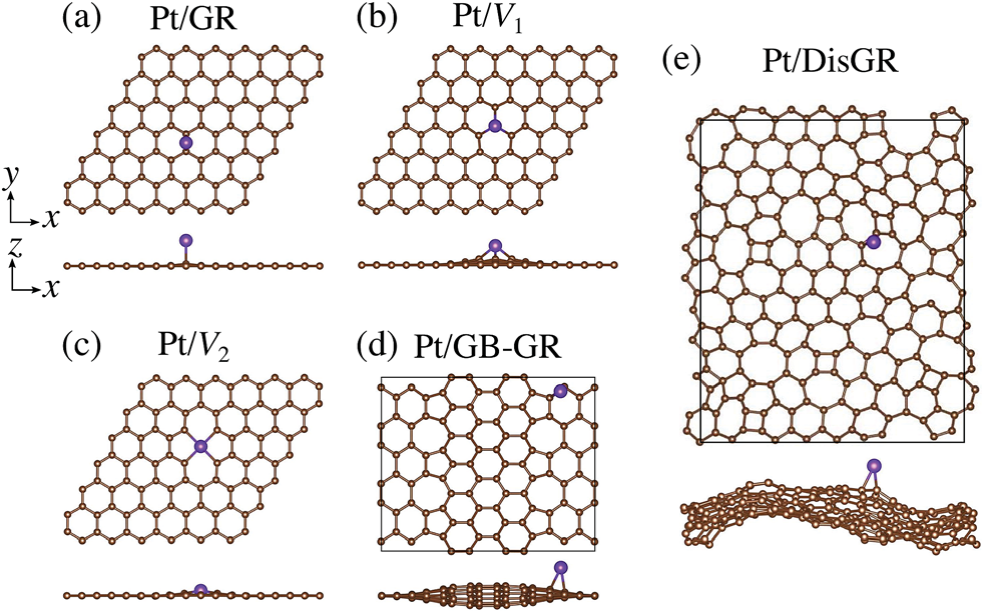
Optimized structures of Pt single-atom adsorption on (a) pristine graphene (GR), (b) graphene with a carbon mono-vacancy (*V*_1_), (c) graphene with a carbon di-vacancy (*V*_2_), (d) grain-boundary graphene (GB-GR), and (e) disorder graphene (DisGR).

We first consider pristine and defective graphene structures and adsorption of single Pt atoms on them as shown in Fig. 1. We calculate the binding energy defined by

1 *E*_b_ = *E*_Pt/G_ − *E*_G_ − *μ*_Pt_,

where *E*_Pt/G_, *E*_G_, and *μ*_Pt_ are the total energy of the adsorption system, the total energy of the substrate, and the chemical potential of Pt, respectively. We use the total energy of an isolated Pt atom for *μ*_Pt_. In Table 1, we summarize the calculated binding energies of a single Pt atom on pristine and defective graphene structures. The binding energy for the pristine graphene is slightly larger than those reported in ref. 26 because of the exchange–correlation functionals used (see Table S1 in the ESI† for the binding energies calculated using the PBE^54^ functional). The Pt single-atom adsorption on graphene with vacancy is significantly stable, because the Pt atom terminates dangling bonds associated with the C vacancy. However, the energies necessary to create the C vacancies (formation energies for C vacancy with respect to the C atom in graphene) are 7.77 eV and 7.86 eV for *V*_1_ and *V*_2_, respectively, and the effective binding energies are +0.07 eV and +0.29 eV, respectively. Thus we conclude that the Pt single-atom adsorption at the C vacancy site is thermodynamically less favorable. The binding energies of a single Pt atom for GB-GR and DisGR are apparently much larger than that for pristine graphene. However, this is because GB-GR and DisGR are less stable and thus more reactive than the pristine one. They are less stable than pristine graphene by 0.17 and 0.39 eV per C atom, respectively.

**Table 1:** Binding energy (*E*_b_) of Pt single-atom adsorption for several graphene based structures

Structure	*E*_b_/eV
Pt/GR	−1.97
Pt/*V*_1_	−7.70
Pt/*V*_2_	−7.57
Pt/GB-GR	−2.55
Pt/DisGR	−2.99

#### 3.1.1 Pristine GNR

**Fig. 2:**
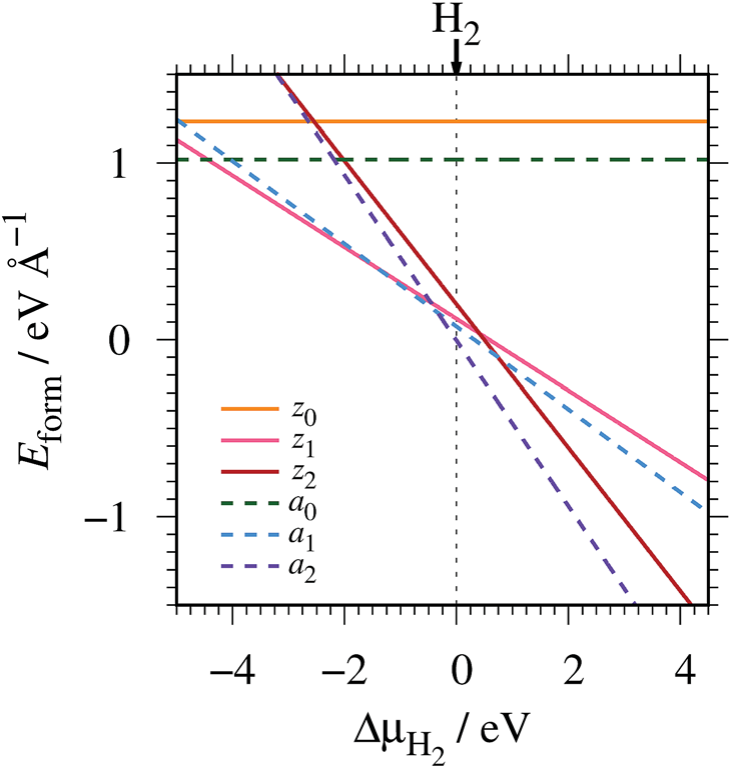
Formation energy of GNR as a function of H_2_ chemical potential. Δ*μ*_H_2__ = *μ*_H_2__ − *E*_H_2__, where *E*_H_2__ is the total energy of a gas-phase H_2_ molecule at 0 K.

We then investigate the most stable GNR by calculating the formation energy defined by2
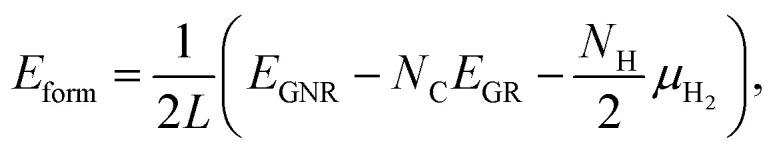
where *E*_GNR_, *E*_GR_, and *μ*_H_2__ are the total energy of GNR, total energy of C atoms in bulk graphene (total energy of graphene per atom), and chemical potential of H_2_ molecule, respectively; *N*_C_ (*N*_H_) is the number of C (H) atoms in GNR; and *L* is the length of the unit cell along the edge. Fig. 2 shows the calculated formation energy as a function of H_2_ chemical potential. It is found that at high H_2_ chemical potential, *a*GNR (*a*_2_) is stable, whereas *z*GNRs are more stable at low H_2_ chemical potential, and non-hydrogenated GNR is unstable in a wide range of H_2_ chemical potential, in good agreement with the previous study.^58^ The positions of the intersections differ from those reported in ref. 58, because the exchange–correlation functionals used are different (see Fig. S1 in the ESI† for comparison of the results obtained using rev-vdW-DF2 and PBE). We also confirm that the stability is insensitive to the GNR width (see Fig. S2 in the ESI†).

To compare the stability of the GNRs with the defective graphene structures, we calculate the formation energy per C atom (replace 2*L* in eqn (2) with *N*_C_). At *μ*_H_2__ = *E*_H_2__, the formation energy per C atom of *z*_0_, *z*_1_, *z*_2_, *a*_0_, *a*_1_, and *a*_2_ are 0.61, 0.06, 0.10, 0.44, 0.03, and ∼0.00 eV, respectively. Thus, although most GNRs considered here are less stable than pristine graphene (*i.e.* most GNRs have positive *E*_form_ at *μ*_H_2__ = *E*_H_2__), hydrogenated GNRs are more stable than defective graphene structures.

### 3.2 Pt single-atom adsorption on *z*GNRs

**Fig. 3:**
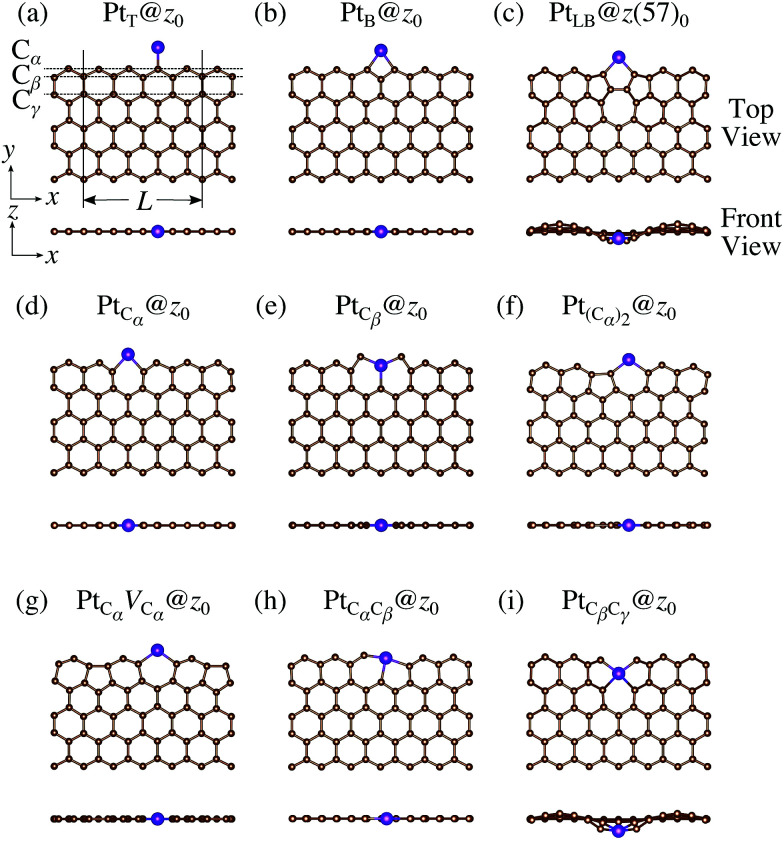
Optimized structures of Pt single-atom adsorption at the edge of non-hydrogenated *z*GNRs. Purple (brown) spheres represent Pt (H) atoms. The first, second, and third outermost carbon atoms are denoted as C_α_, C_β_, and C_γ_, respectively. *L* indicates the periodicity of the ribbons along the edge direction.

We consider Pt single-atom adsorption on non-hydrogenated *z*GNR (*z*_0_). We systematically construct the adsorption configurations with and without defects, and label them according to the Kröger–Vink notation,^65^ as shown in Fig. 3. We first consider the Pt single-atom adsorption at the top- and bridge-site of perfect *z*_0_, denoted as Pt_T_@*z*_0_ (Fig. 3(a)) and Pt_B_@*z*_0_ (Fig. 3(b)), respectively. Second, we introduce a Stone–Wales (SW) defect at the edge of *z*_0_, denoted as *z*(57)_0_, and a Pt atom adsorbed at the long-bridge (LB) site of the SW defect, denoted as Pt_LB_@*z*(57)_0_ (Fig. 3(c)). We introduce substitutional Pt with single edge carbon atoms (C_α_, C_β_) denoted as Pt_C_α__@*z*_0_ (Fig. 3(d)) and Pt_C_β__@*z*_0_ (Fig. 3(e)), respectively. We also consider two outermost carbon vacancies (*V*_C_), and obtained the Pt substituted with two *V*_C_'s (Pt(C_α_)@*z*_0_, Fig. 3(f)) and a complex of a substitutional Pt and *V*_C_ (Pt_C_α__*V*_C_α__@*z*_0_, Fig. 3(g)). Finally we consider Pt configurations with a divacancy formed with the outermost and second outermost atoms and that with second and third outermost C atoms (Pt_C_α_C_β__@*z*_0_ (Fig. 3(h)) and Pt_C_β_C_γ__@*z*_0_ (Fig. 3(i), respectively).

For the optimized structures with a single Pt atom at the edge of *z*_0_, we calculate the Gibbs free energy defined by

**Fig. 4:**
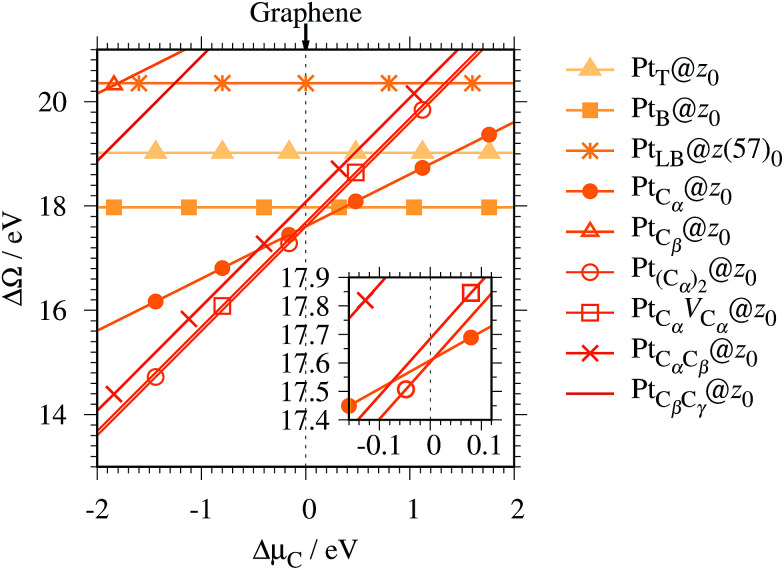
The Gibbs free energy for the pristine GNRs as a function of C chemical potentials. Δ*μ*_C_ = *μ*_C_ − *E*_GR_ is the C chemical potential referenced to the total energy of graphene at 0 K (*E*_GR_).

3 Δ*Ω*(*μ*_C_) = *E*_Pt@GNR_ − *E*_ref_ − *μ*_Pt_ − Δ*N*_C_*μ*_C_,

where *E*_Pt@GNR_ and *E*_ref_ are the total energies of adsorbed and reference systems, respectively; Δ*N*_C_ is the difference of the number of C atoms from the reference system; and *μ*_Pt_ and *μ*_C_ are the chemical potentials of Pt and C, respectively. Here we choose pristine graphene as the reference (*E*_ref_ = *E*_GR_ × *N*_C_, where *N*_C_ is the number of C atoms in the perfect GNR). *μ*_Pt_ is chosen to be the total energy of an isolated Pt atom (*E*_Pt_) and *μ*_C_ varies around the chemical potential of graphene. Fig. 4 shows the Gibbs free energy as a function of C chemical potential for nine structures considered in this work, and those at *μ*_C_ = *E*_GR_ (the system is in equilibrium with graphene) are summarized in Table 2. We can see that all the structures have large positive Gibbs free energies at *μ*_C_ = *E*_GR_, suggesting that Pt single-atom adsorption at the edges of *z*_0_ is thermodynamically unstable. Among the Pt adsorption structures on *z*_0_, Pt_C_α__@*z*_0_ is the most favorable configuration. However, the calculated CLS for Pt_C_α__@*z*_0_ is too small compared with the experimental value of (+2.0 ± 0.4) eV,^42^ suggesting that this adsorption configuration is unlikely. On the other hand, Pt_C_β__@*z*_0_ and Pt_C_β_C_γ__@*z*_0_ show a relatively large CLS of +1.44 and +1.67 eV, respectively. However, their Gibbs free energies are significantly large, ruling out these configurations. Thus, we conclude that Pt single-atom adsorption on the non-hydrogenated GNR is unlikely under the equilibrium conditions.

**Table 2:** Gibbs free energy (Δ*Ω*) and core level shift (CLS) of Pt atom@*z*GNRs at *μ*_C_ = *E*_GR_ and *μ*_H_ = *E*_H_2__

	Δ*Ω*(*E*_GR_,*E*_H_2__)/eV			CLS/eV		
	*z*_0_	*z*_1_	*z*_2_	*z*_0_	*z*_1_	*z*_2_
Pt_T_	+19.02	+0.15	+1.61	−0.56	−0.93	−1.30
Pt_B_	+17.98	+1.57	+2.57	+0.44	+0.35	+0.35
Pt_LB_^a^	+20.35	+3.88	+4.86	+0.44	+0.44	+0.29
Pt_C_α__	+17.61	−0.25	+1.92	+0.50	+1.43	+1.20
Pt_C_β__	+22.16	+1.89	+0.51	+1.44	+1.37	+1.42
Pt_(C_α_)_	+17.60	+1.79	+2.98	−0.29	+0.24	+0.48
Pt_C_α__*V*_C_α__	+17.69	+2.02	+3.33	−0.42	+0.54	+0.88
Pt_C_α_C_β__	+18.08	+0.14	+1.62	+0.81	+0.81	+0.52
Pt_C_β_C_γ__	+22.87	+2.11	+1.21	+1.67	+0.82	+1.11
Expt	—	—	—	+2.0 ± 0.4^b^		

a Pt_LB_ should be adsorbed at the edge of *z*(57)_*n*_.

b Taken from ref. 42.

**Fig. 5:**
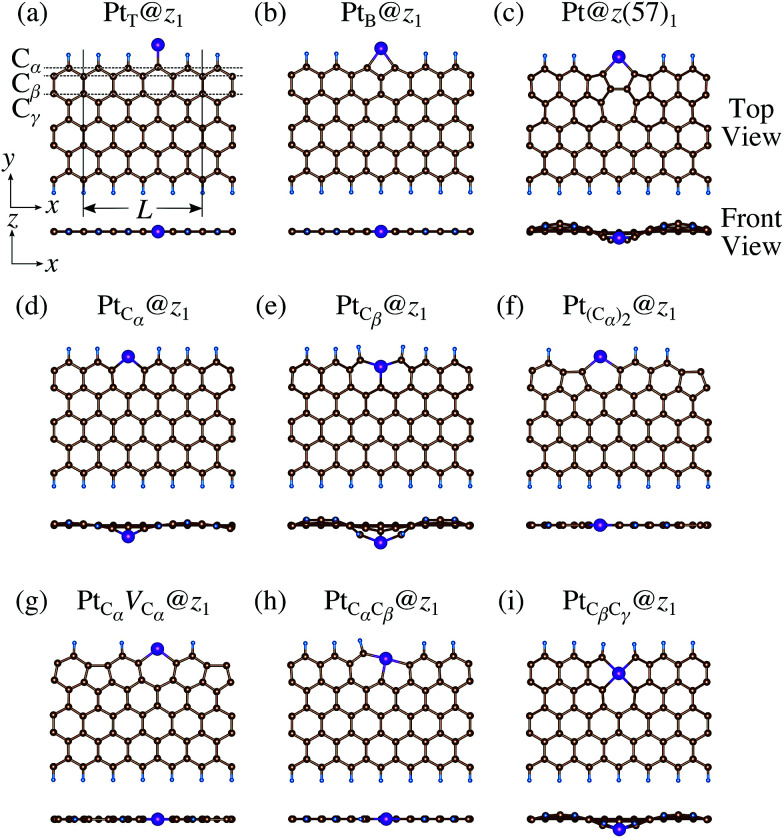
Optimized structures of Pt single-atom adsorption at the edges of mono-hydrogenated *z*GNRs. Purple, brown, and light blue spheres represent Pt, C, and H atoms, respectively. The first, second, and third outermost C atoms are denoted as C_α_, C_β_, and C_γ_, respectively. *L* indicates the periodicity of the ribbons along the edge direction.

**Fig. 6:**
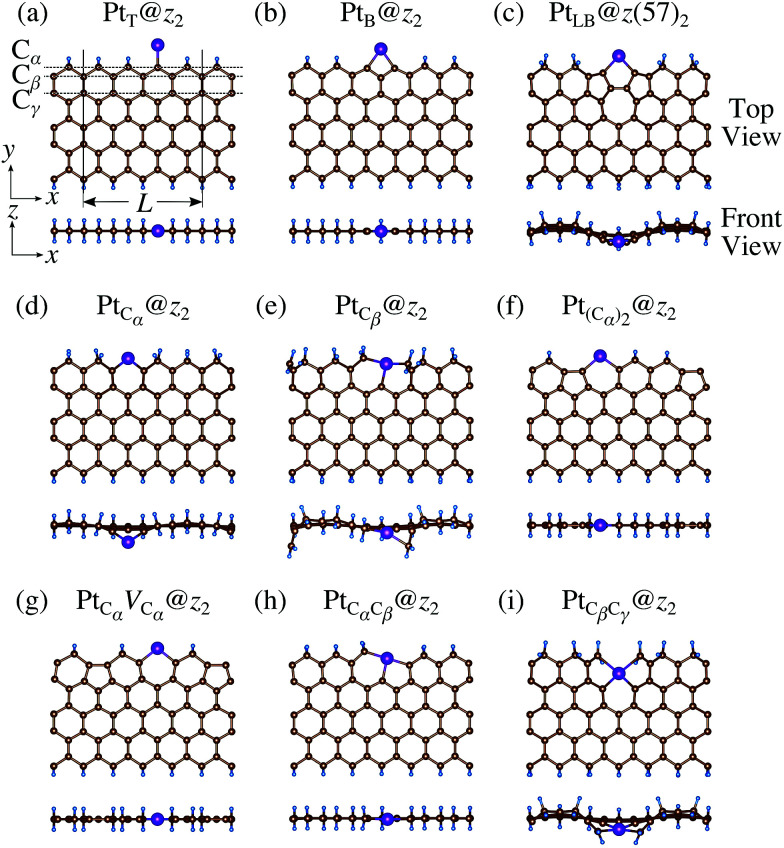
Optimized structures of Pt single-atom adsorption at the edges of di-hydrogenated *z*GNRs. Purple, brown, and light blue spheres represent Pt, C, and H atoms, respectively. The first, second, and third outermost C atoms are denoted as C_α_, C_β_, and C_γ_, respectively. *L* indicates the periodicity of the ribbons along the edge direction.

**Fig. 7:**
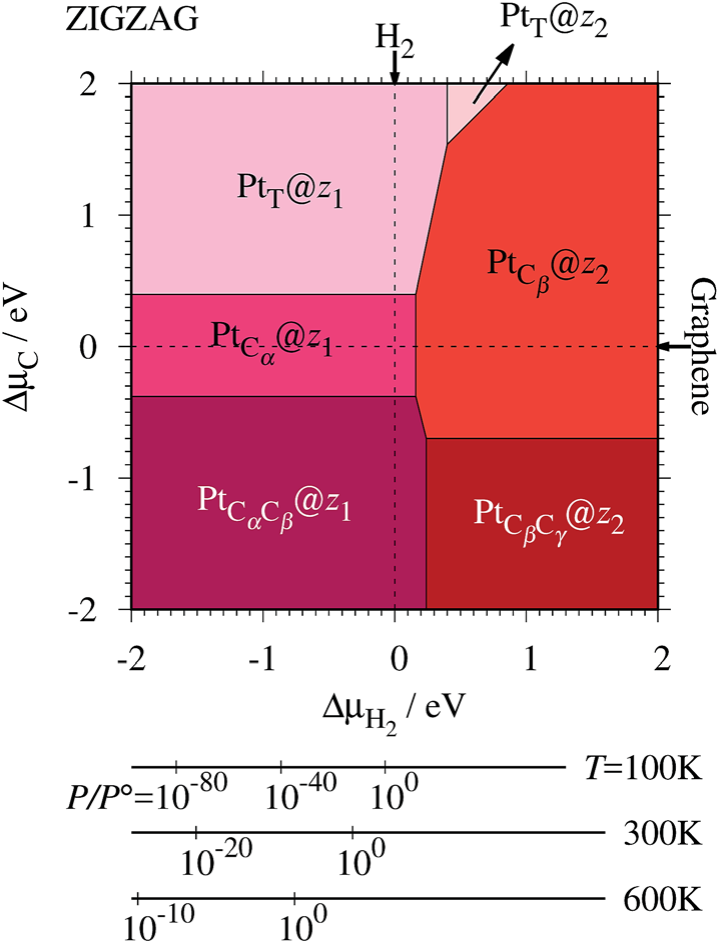
The Gibbs free energy for Pt single-atom adsorption at the edge of *z*GNRs as a function of C and H_2_ chemical potentials. Δ*μ*_C_ = *μ*_C_ − *E*_GR_ and Δ*μ*_H_2__ = *μ*_H_2__ − *E*_H_2__ are C and H_2_ chemical potentials referenced to the total energies of gas-phase H_2_ and graphene, respectively. The bottom axes show the corresponding H_2_ chemical potentials at the absolute temperature *T* and partial pressure *P* (with *P°* = 1 atm), *μ*_H_2__ = *H°*(*T*) − *H°*(0) − *TS°*(*T*) + *k*_B_*T* ln(*P*/*P°*), where the enthalpy *H°* and the entropy *S°* are obtained from ref. 66.

We then investigate the stability of Pt single-atom adsorption on mono- and di-hydrogenated *z*GNRs (*z*_1_ and *z*_2_). The structures are similar to those adopted for non-hydrogenated *z*GNRs as shown in Fig. 5 and 6 for *z*_1_ and *z*_2_, respectively. We optimized all the structures and calculated the Gibbs free energy given by4
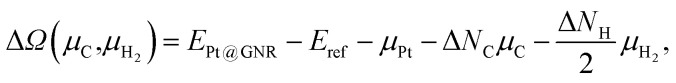
where *E*_Pt@GNR_ (*E*_ref_) is the total energy of the adsorbed system (reference system), *μ*_Pt_ = *E*_Pt_, Δ*N*_C_, and Δ*N*_H_ are the difference of numbers of C and H atoms from the reference system, respectively, and *μ*_C_ and *μ*_H_2__ are chemical potentials of C atom and H_2_ molecule, respectively. As in the case of *z*_0_, pristine graphene is chosen as the reference. We calculate Δ*Ω* for *z*_0_, *z*_1_, and *z*_2_ as a function of *μ*_H_2__ and *μ*_C_ and generate the phase diagram as shown in Fig. 7. We also calculate Δ*Ω* at *μ*_C_ = *E*_GR_ and *μ*_H_2__ = *E*_H_2__ for the structures considered, where *E*_H_2__ is the total energy of an isolated H_2_ molecule. The results are summarized in Table 2 along with the calculated CLSs for each structure. As expected, single Pt atom adsorbed non-hydrogenated *z*GNR is unstable and does not appear in the phase diagram. We find that Pt_C_α__@*z*_1_ only exhibits negative Δ*Ω* at *μ*_C_ = *E*_GR_ and *μ*_H_2__ = *E*_H_2__, suggesting that this structure is thermodynamically stable under these conditions. Furthermore, the calculated CLS for this structure is in reasonable agreement with the experiment. Among the hydrogenated GNR structures, calculated CLSs for Pt_C_β__@*z*_1_ and Pt_C_α__@*z*_2_ are also reasonable. However, they show positive Δ*Ω*'s, implying that these structures are less likely than Pt_C_α__@*z*_1_.

### 3.3 Pt single-atom adsorption on *a*GNRs

**Fig. 8:**
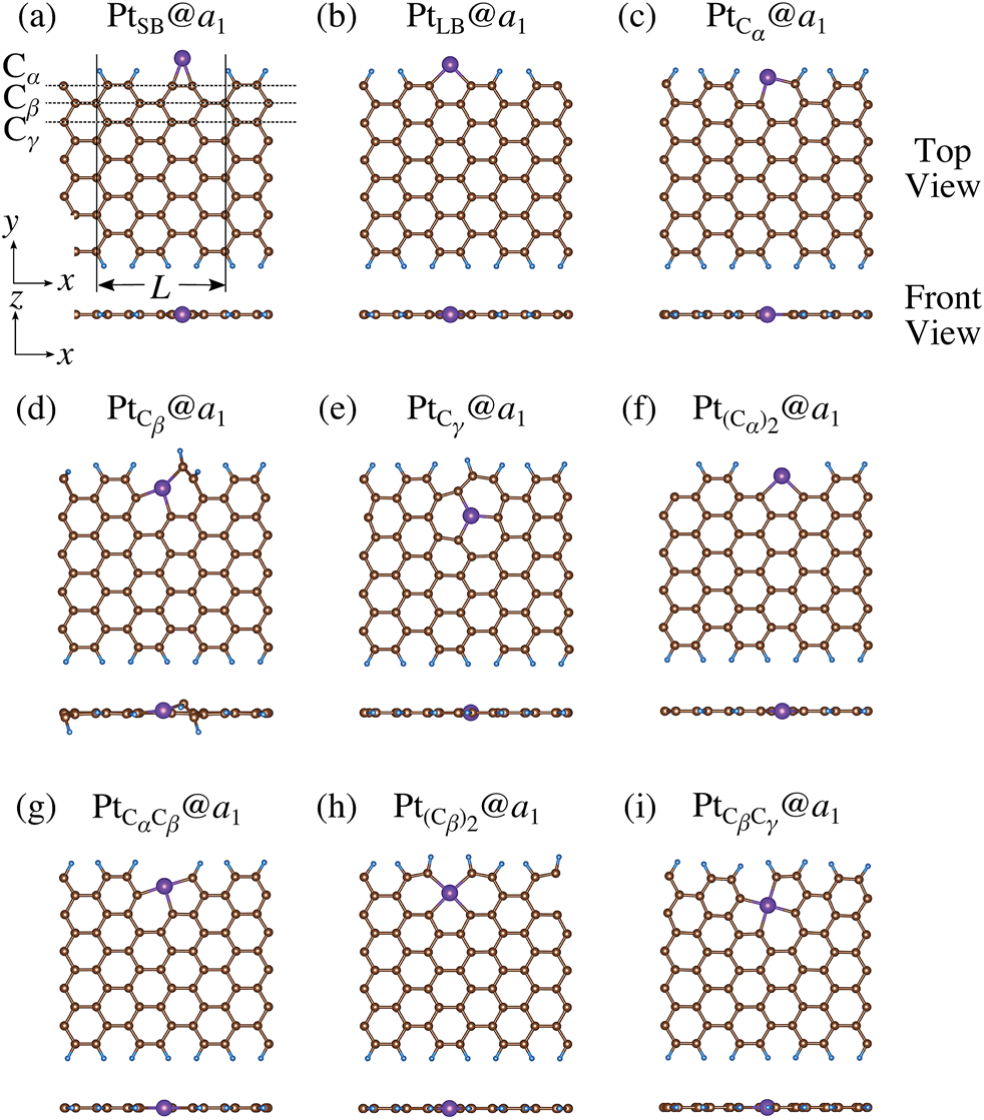
Optimized structures of Pt single-atom adsorption at the edges of mono-hydrogenated *a*GNRs. Purple, brown, and light blue spheres represent Pt, C, and H atoms, respectively. The first, second, and third outermost C atoms are denoted as C_α_, C_β_, and C_γ_, respectively. *L* indicates the periodicity of the ribbons along the edge direction.

**Fig. 9:**
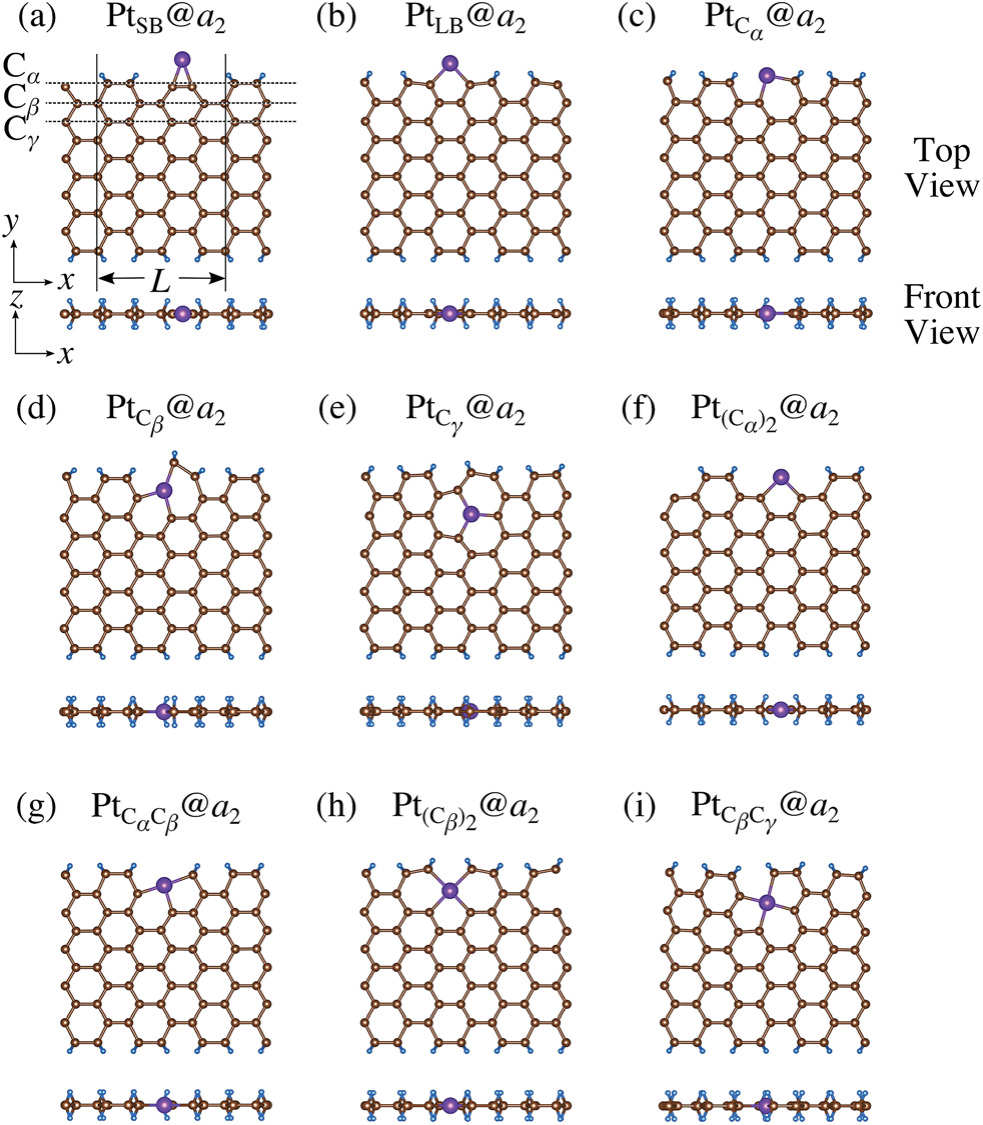
Optimized structures of Pt single-atom adsorption at the edges of di-hydrogenated *a*GNRs. Purple, brown, and light blue spheres represent Pt, C, and H atoms, respectively. The first, second, and third outermost C atoms are denoted as C_α_, C_β_, and C_γ_, respectively. *L* indicates the periodicity of the ribbons along the edge direction.

**Fig. 10:**
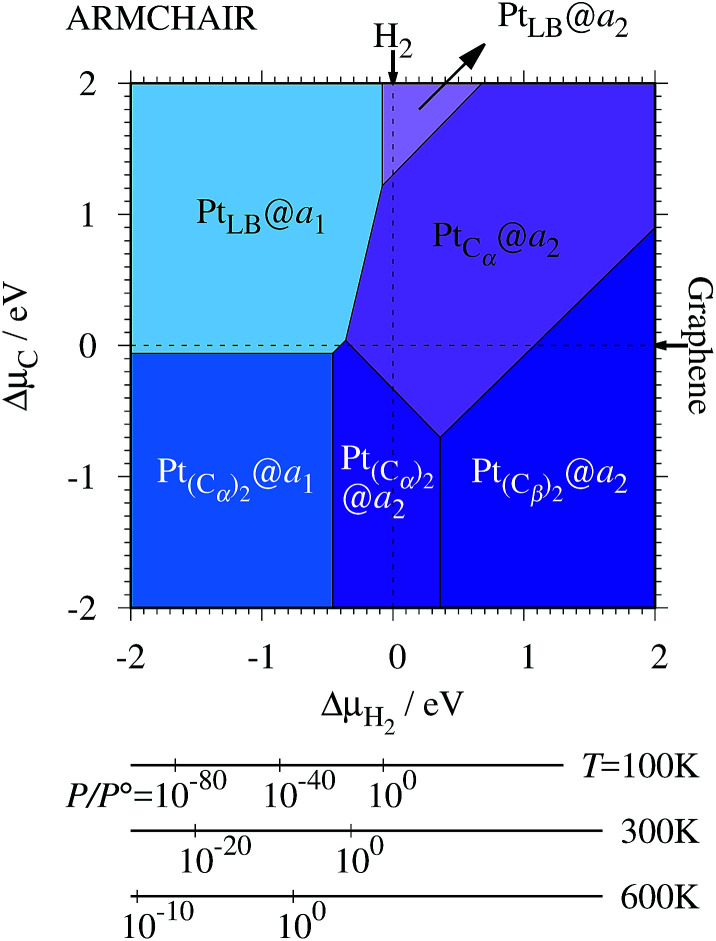
The Gibbs free energy for Pt single-atom adsorption at the edges of *a*GNRs as a function of C and H_2_ chemical potentials. Δ*μ*_C_ = *μ*_C_ − *E*_GR_ and Δ*μ*_H_2__ = *μ*_H_2__ − *E*_H_2__ are C and H_2_ chemical potentials referenced to the total energies of gas-phase H_2_ and graphene, respectively. The bottom axes show the corresponding H_2_ chemical potentials at the absolute temperature *T* and partial pressure *P* (with *P°* = 1 atm), *μ*_H_2__ = *H°*(*T*) − *H°*(0) − *TS°*(*T*) + *k*_B_*T* ln(*P*/*P°*), where the enthalpy *H°* and the entropy *S°* are obtained from ref. 66.

We investigate the Pt single-atom adsorption at the edge of mono- and di-hydrogenated *a*GNRs. We do not consider non-hydrogenated *a*GNRs as the non-hydrogenated structures are unstable as observed in the case of *z*GNR. We consider the following structures: Pt adsorbed at short-bridge (SB) and long-bridge (LB) sites (Fig. 8(a) and (b), respectively); substitutional Pt with single C atom respectively (Fig. 8(c)–(e)); substitutional Pt with two C atoms (Fig. 8(f)–(i)). We construct similar structures for dihydrogenated *a*GNR (Fig. 9). The structures are fully optimized and the Gibbs free energies are calculated according to eqn (4). The phase diagram for *a*GNR is shown in Fig. 10, and Δ*Ω*'s at *μ*_C_ = *E*_GR_ and *μ*_H_2__ = *E*_H_2__ for different adsorption configurations are summarized in Table 3. We find that Pt_C_α__@*z*_2_ is the most stable at *μ*_C_ = *E*_GR_ and *μ*_H_2__ = *E*_H_2__ with reasonable CLS, suggesting that this structure is the most likely candidate under these conditions. Furthermore, the absolute value of calculated Δ*Ω* is much larger than that of the most stable *z*GNR, suggesting that Pt adsorbed at *a*GNR is thermodynamically more stable than Pt adsorbed at *z*GNR. We also find that Pt_C_β__@*a*_1_, Pt_C_γ__@*a*_1_, and Pt_C_γ__@*a*_2_ show relatively large positive CLSs. However, they show large positive Δ*Ω* at *μ*_C_ = *E*_GR_ and *μ*_H_2__ = *E*_H_2__ and therefore appear in the phase diagram (Fig. 10). In particular the latter two show large positive Δ*Ω* and are less likely.

**Table 3:** The Gibbs free energy (Δ*Ω*) for Pt atom adsorption at *a*GNRs at *μ*_C_ = *E*_GR_ and *μ*_H_ = *E*_H_2__ and the corresponding core level shift (CLS)

	Δ*Ω*(*E*_GR_,*E*_H_2__)/eV		CLS/eV	
	*a*_1_	*a*_2_	*a*_1_	*a*_2_
Pt_SB_	+0.67	−0.32	+0.63	+0.60
Pt_LB_	−0.69	−0.94	+0.44	+0.77
Pt_C_α__	−0.35	−2.24	+1.33	+1.33
Pt_C_β__	+0.91	−0.02	+1.56	+0.82
Pt_C_γ__	+4.15	+3.00	+2.36	+2.33
Pt_(C_α_)_2__	−0.57	−1.90	+0.17	+0.51
Pt_C_α_C_β__	+0.33	−1.40	+0.53	+0.77
Pt_(C_β_)_2__	+0.51	−1.17	−0.11	−0.74
Pt_C_β_C_γ__	+1.48	−0.44	+0.05	−0.03
Expt.	—	—	+2.0 ± 0.4^a^	

a Taken from ref. 42.

### 3.4 Impact of the substrate

**Fig. 11:**
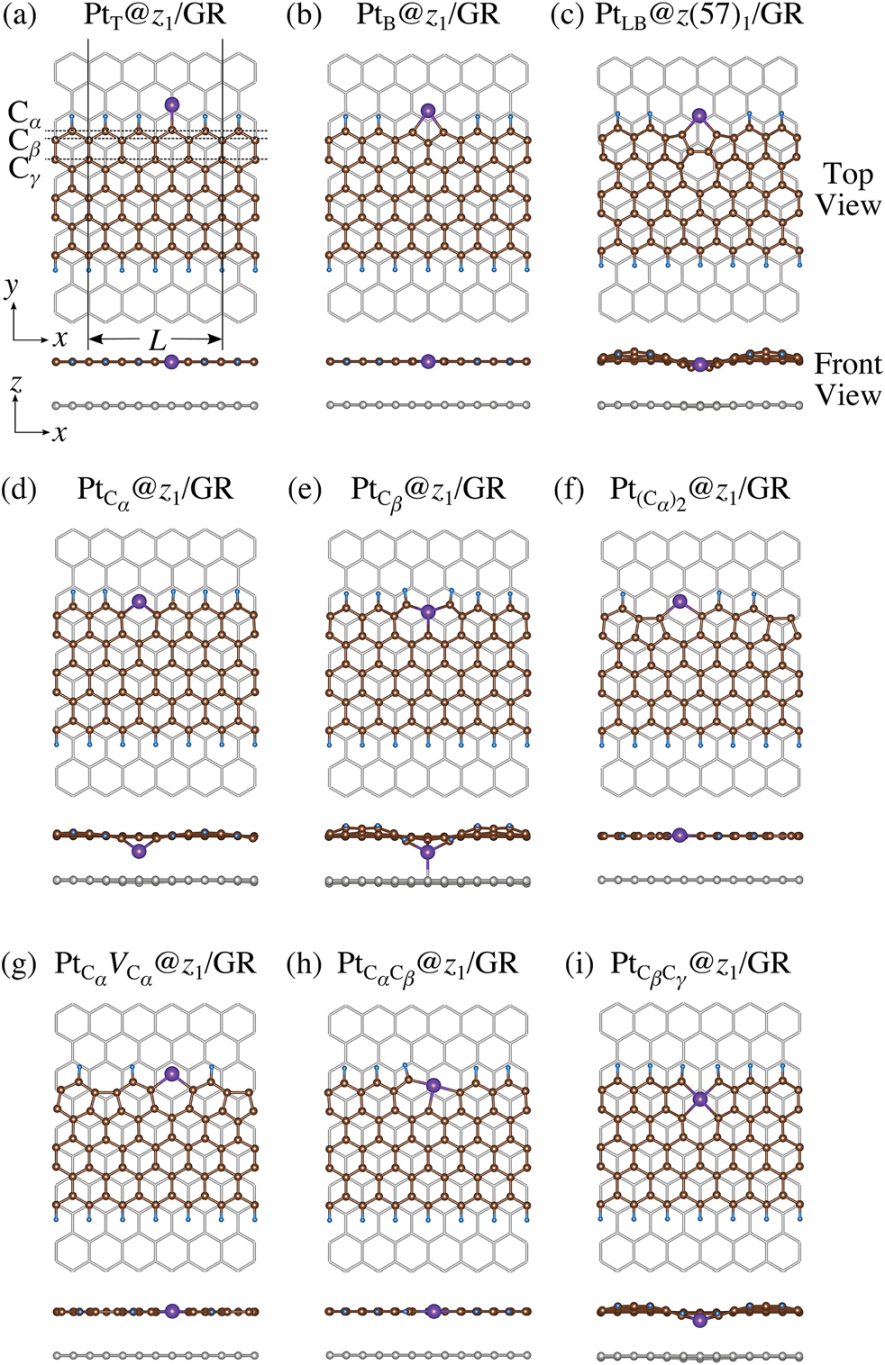
Optimized structures of Pt single-atom adsorption at the edges of mono-hydrogenated *z*GNRs with a graphene substrate. Purple, brown, and light blue spheres represent Pt, C, and H atoms, respectively and the grey honeycomb corresponds to the graphene layer. The first, second, and third outermost C atoms are denoted as C_α_, C_β_, and C_γ_, respectively. *L* indicates the periodicity of the ribbons along the edge direction.

Here we examine the effect of the substrate, as in the experiment,^42^ Pt single-atom adsorption has been observed at the step edge of graphite, and the graphene underneath may play some role. We perform the structural optimization with the graphene substrate and calculate the CLS of Pt single-atom at the edge of *z*GNR. We adopt the adsorption structures with *z*_1_ and introduced graphene underneath as shown in Fig. 11. We optimize the structures and calculate the binding energy defined by5
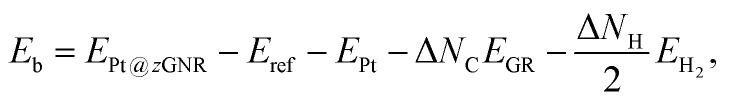
where *E*_Pt@*z*GNR_ is the total energies of Pt adsorbed at *z*GNR (with and without the graphene substrate) and *E*_ref_ is the total energy of the reference system, where we use perfect *z*_1_ and perfect *z*_1_ on graphene (*z*_1_/GR) as references for the binding energies for a Pt single-atom at *z*_1_ and at *z*_1_/GR, respectively. Calculated binding energies and corresponding CLSs are summarized in Table 4. We can see that the difference of the binding energies with and without the substrate is insignificant, and in most cases, the substrate plays a role in stabilizing the Pt single-atom adsorption, except for Pt_C_β__@*z*_1_. The most stable adsorption structure (Pt_C_α__@*z*_1_) is unchanged and the binding energy difference is 0.16 eV upon inclusion of the substrate. The substrate does have an insignificant impact on the calculated core level shift, especially for the most stable Pt_C_α__@*z*_1_. The change is −0.19 eV and the conclusion is unaltered.

**Table 4:** The binding energy (*E*_b_) and the core level shift (CLS) of Pt single-atom adsorption at the edges of *z*_1_ with and without the substrate graphene (GR)

	*E*_b_/eV		CLS/eV	
	w/o GR	w/GR	w/o GR	w/GR
Pt_T_@*z*_1_	−2.19	−2.32	−0.93	−0.94
Pt_B_@*z*_1_	−0.77	−0.88	+0.35	+0.34
Pt_LB_@*z*(57)_1_	+1.55	+1.70	+0.44	+0.51
Pt_C_α__@*z*_1_	−2.58	−2.74	+1.43	+1.24
Pt_C_β__@*z*_1_	−0.44	−0.33	+1.37	+1.99
Pt(C_α_)@*z*_1_	−0.54	−0.58	+0.24	+0.21
Pt_C_α__*V*_C_α__@*z*_1_	−0.32	−0.36	+0.54	−0.50
Pt_C_α_C_β__@*z*_1_	−2.19	−2.17	+0.81	+0.81
Pt_C_β_C_γ__@*z*_1_	−0.22	−0.01	+0.82	+0.78
Expt	—	—	+2.0 ± 0.4^a^	

a Taken from ref. 42.

## 4 Discussion

**Fig. 12:**
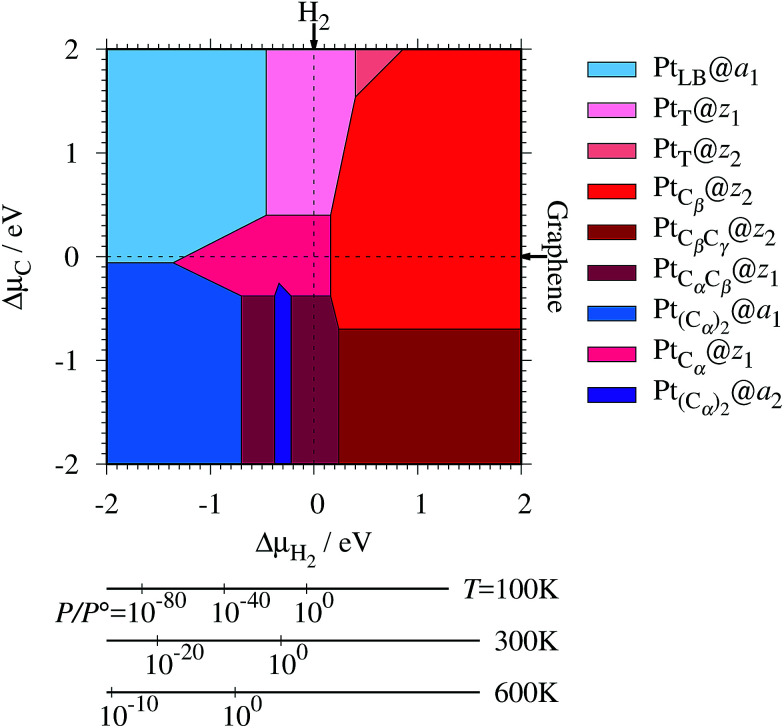
The binding energy for Pt single-atom adsorption at the edge of GNRs as a function of C and H_2_ chemical potentials. The chemical potentials of C (Δ*μ*_C_) and H_2_ (Δ*μ*_H_2__) are referenced to the total energies of graphene and gas-phase H_2_ molecule at 0 K, respectively. The bottom axes show the corresponding H_2_ chemical potentials at the absolute temperature *T* and partial pressure *P* (with *P°* = 1 atm), *μ*_H_2__ = *H°*(*T*) − *H°*(0) − *TS°*(*T*) + *k*_B_*T* ln(*P*/*P°*), where the enthalpy *H°* and the entropy *S°* are obtained from ref. 66.

Experimentally, both zigzag and armchair edges coexist depending on the environment. Thus, it is desirable to compare the stability of single Pt atoms adsorbed at the edges of *z*GNR and *a*GNR on the same footing, and investigate the favorable adsorption site when both edges are exposed. For this purpose, we define the binding energy of a single Pt atom as6
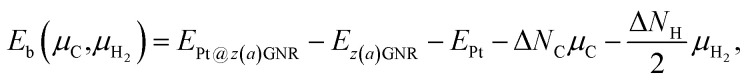
where *E*_Pt@*z*(*a*)GNR_ and *E*_*z*(*a*)GNR_ are total energies of Pt adsorbed *z*GNR (*a*GNR) and pristine *z*GNR (*a*GNR), respectively. We note that the total energy of the most stable form of GNR at a given *μ*_H_2__ is used as a reference (*E*_*z*(*a*)GNR_, see also Fig. 2). *E*_b_ is an indicator of the strength of Pt single-atom adsorption and can be used to discuss the stability of Pt when both *z*GNR and *a*GNR are present. Calculated *E*_b_'s as a function of *μ*_C_ and *μ*_H_2__ (phase diagram) are shown in Fig. 12 and those at *μ*_C_ = *E*_GR_ and *μ*_H_2__ = *E*_H_2__ are summarized in Table 5. We find that although *a*GNRs and Pt adsorbed *a*GNRs are thermodynamically more stable than *z*GNRs in terms of the Gibbs free energy (see Tables 3 and 2), Pt adsorption at the edges of *z*GNRs is more favorable. This suggests that when armchair and zigzag edges coexist, single Pt atoms prefer to adsorb at the zigzag edge. At around *μ*_C_ = *E*_GR_, Pt_C_α__@*z*_1_ and Pt_C_β__@*z*_2_ are the most likely adsorption structures. In particular, the calculated CLS for the former agrees reasonably with the experimental one, and thus, we conclude that Pt_C_α__@*z*_1_ is the most probable structure. Note that at −0.38 eV ≤ Δ*μ*_H_2__ ≤ −0.22 eV, Pt(C_α_)_2_@*a*_2_ appears as a stable structure among Pt_C_α_C_β__@*z*_1_. This narrow *a*_2_ region corresponds to the region where intersections of formation energies are found (Fig. 2) and the stable (unstable) phase is determined by a subtle energy balance. As a result, *a*_2_ becomes unstable, *i.e.*, more reactive, and Pt(C_α_)_2_@*a*_2_ emerges as a stable adsorption structure. To determine the precise stability (boundary) when the different phases compete, however, more accurate and precise calculation of total energy is required. Nevertheless, the stability at around *μ*_C_ = *E*_GR_ and *μ*_H_2__ = *E*_H_2__ is unaffected, and we pursue this in future work. Very recently, Yamazaki *et al.* proposed the threefold-coordinated Pt atom at the edges of graphene flakes based on X-ray photoelectron spectroscopy and DFT calculations,^42^ which contradicts our finding that the thermodynamically most stable configuration (PtCa@z_1_) has a twofold-coordinated Pt atom. On the other hand, the metastable Pt_C_β__@*z*_1_ in our study, which has a threefold-coordinated Pt atom, gives relatively large binding energy and reasonable CLS, and we do not rule out the possibility of the threefold-coordinated Pt atom at the edge at this point. To resolve the discrepancy, we may need to employ the state-of-the-art theoretical method to calculate the absolute binding energy,^67^ and to investigate the models for the graphene edge, because we and Yamazaki *et al.* use different models (GNR and graphene flakes, respectively) for the graphene edge. Nevertheless, we can conclude that the Pt single-atom adsorption takes place dominantly at the edge of graphene, and determination of a more precise position and CLS will be done in the near future.

**Table 5:** The binding energies (*E*_b_) of the favorable Pt single-atom adsorption configurations at *μ*_C_ = *E*_GR_ and *μ*_H_ = *E*_H_2__

Configuration	*E*_b_/eV
Pt_T_@*z*_1_	−2.19
Pt_C_α__@*z*_1_	−2.58
Pt_C_α_C_β__@*z*_1_	−2.19
Pt_T_@*z*_2_	−0.72
Pt_C_β__@*z*_2_	−1.82
Pt_C_β_C_γ__@*z*_2_	−1.13
Pt_LB_@*a*_1_	−0.61
Pt_(C_α_)_2__@*a*_1_	−0.49
Pt_(C_α_)_2__@*a*_2_	−1.82

Here, let us discuss the oxidation state of a single Pt atom, as it is crucial to the understanding of its catalytic activity. We calculated the density of states projected on the atomic orbitals of Pt for Pt_C_α__@*z*_1_ (Fig. S3 in the ESI†), which gives CLS in good agreement with the experimental value. We found that the Pt d states hybridize with the GNR state. In particular, the Pt d_*xy*_ state hybridizes strongly with the C sp state and forms a fully unoccupied antibonding state, resulting in the formal oxidation state of 2+. We also performed the Bader charge analysis (Table S2 in the ESI†), and found that it is not straightforward to assign the oxidation state of Pt only from the Bader charge analysis, especially those at the edges, because of the strong hybridization of Pt *d* states with GNR.

## 5 Conclusions

We present a systematic density functional theory-based thermodynamics study of Pt single-atom adsorption on graphene. We find that single Pt atoms adsorb more preferably at the graphene edge than on the bulk. Although pristine *a*GNR is thermodynamically more stable than *z*GNR under a wide range of hydrogen pressure, single Pt atoms preferably adsorb at the edge of hydrogenated *z*GNR. The calculated core level shifts for the stable structures are in reasonable agreement with the experiment, supporting our findings. Our study will serve as a basis for further investigation of the catalytic activity of single-atom catalysts based on single Pt atoms and graphene based nanostructures.

## Conflicts of interest

There are no conflicts to declare.

## Supplementary Material

NA-001-C8NA00236C-s001
