# Supplementary material for: Platinum single-atom adsorption on graphene: a density functional theory study
Source: Nanoscale Adv. 2019 Jan 8;1(3):1165–74. doi: 10.1039/c8na00236c (PMC9417699; doi:10.1039/c8na00236c)
Supplement: NA-001-C8NA00236C-s001 [file NA-001-C8NA00236C-s001.pdf]

Electronic Supplementary Material (ESI) for Nanoscale Advances.
This journal is © The Royal Society of Chemistry 2018

Supplementaly Electronic Information: Platinum Single-atom Adsorption on Graphene: A Density Functional Theory Study

Sasfan Arman Wella,[1,2] Yuji Hamamoto,[1] Suprijadi,[2]
Yoshitada Morikawa,[1,3] and Ikutaro Hamada[1]

[1] *Department of Precision Science and Technology, Graduate School of Engineering, Osaka University, 2-1 Yamada-oka Suita, Osaka 565-0871, Japan*

[2] *Department of Physics, Faculty of Mathematics and Natural Sciences, Institut Teknologi Bandung, Jalan Ganesha 10, Bandung 40132, Indonesia*

[3] *Research Center for Ultra-Precision Science and Technology, Graduate School of Engineering, Osaka University, 2-1 Yamada-oka, Suita, Osaka 565-0871, Japan*

I. PRELIMINARY

In this Electronic Supplementary Information, we present functional dependence of the biding energy of the single Pt atom on graphitic materials and the GNR stability, the width dependence of GNRs, density of states projected onto atomic orbitals for $\mathrm{Pt}_{\mathrm{C}_\alpha}@z_1$, which gives the core level shift agreeing with the experiment, Bader charge of the single Pt atom adsorbed on graphene and graphene nanoribbons (GNRs) obtained using the Bader charge analysis code[1–4].

II. FUNCTIONAL DEPENDENCE OF THE BINDING ENERGIES OF THE SINGLE PT ATOM ON GRAPHITIC MATERIALS

TABLE S1. Binding energies of the single Pt atom on graphitic materials calculated using the Perdew-Burke-Ernzerhof functional. The unit of energy is eV.

Structure	E_b
Pt/GR	−1.61
Pt/V_1	−7.35
Pt/V_2	−7.29
Pt/GB-GR	−2.20
Pt/DisGR	−2.69

III. FUNCTIONAL DEPENDENCE OF THE GRAPHENE NANORIBBON STABILITY

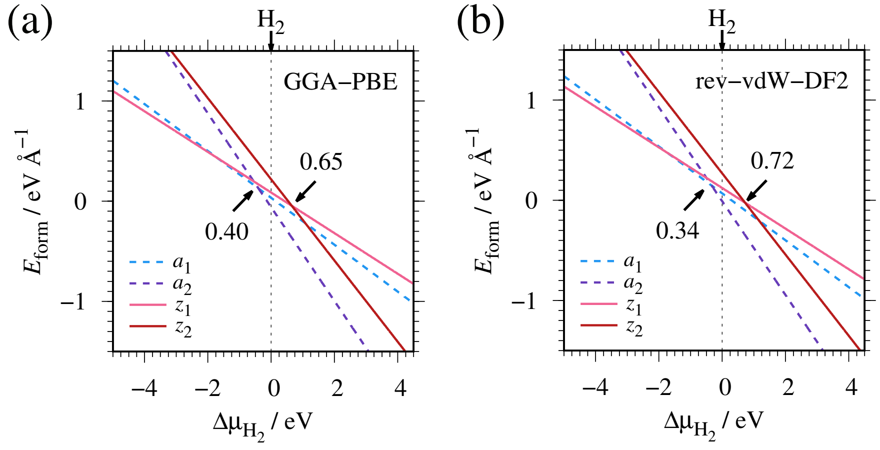

FIG. S1. Formation energies (E_{form}) of armchair and zigzag graphene nanoribbon as a function of chemical potential of hydrogen molecule μ_{H_2}, calculated using (a) PBE and (b) rev-vdW-DF2. 20-aGNR and 10-zGNR were used.

IV. WIDTH DEPENDENCE OF THE GRAPHENE NANORIBBON STABILITY

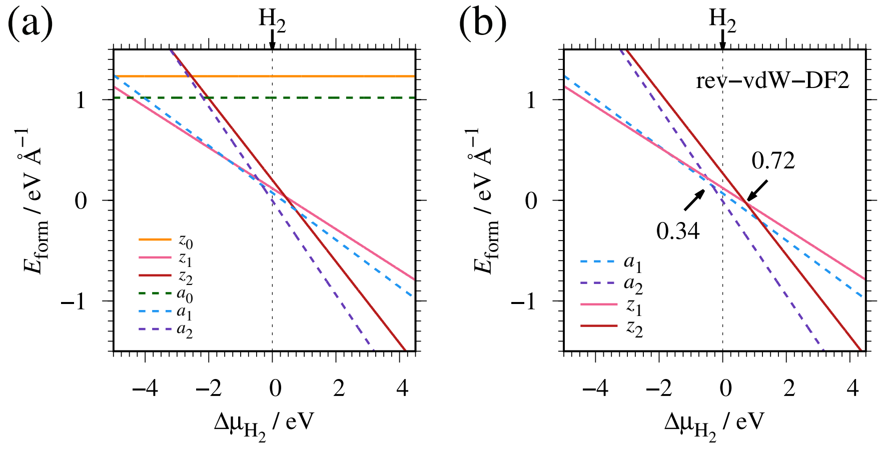

FIG. S2. Formation energies (E_{form}) of armchair and zigzag graphene nanoribbon as a function of chemical potential of hydrogen molecule μ_{H_2}, calculated with (a) 10-aGNR and 5-zGNR and (b) 20-aGNR and 10-zGNR.

V. DENSITY OF STATES

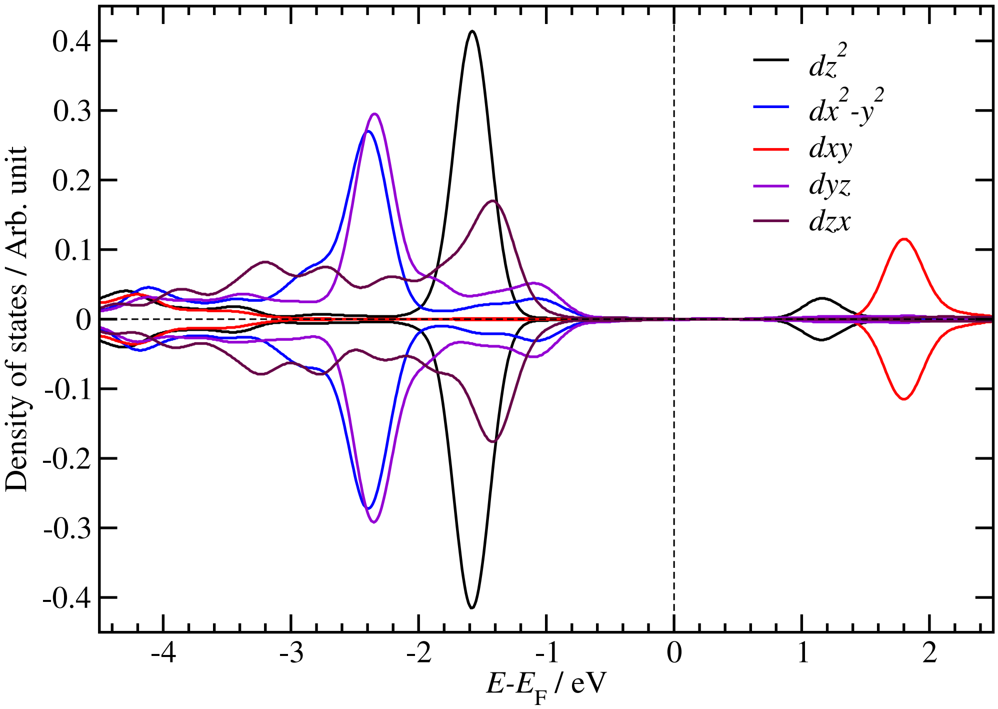

FIG. S3. Densities of states projected onto the Pt d orbitals for $\mathrm{Pt}_{\mathrm{C}_\alpha}@z_1$. The energy origin is set to the Fermi level (E_F).

VI. BADER CHARGE

TABLE S2. Bader charge (Q_{tot}) of the Pt atom and the deviation from the charge of the isolated atom (ΔQ_{tot}) along with the calculated core level shift (CLS).

Structure	Q_{tot}	ΔQ	CLS
Pt/GR	9.94	−0.06	
Pt/V_1	9.51	−0.49	
Pt/V_2	9.24	−0.76	
$\mathrm{Pt_T}@z_0$	9.80	−0.20	−0.56
$\mathrm{Pt_B}@z_0$	9.69	−0.31	0.44
$\mathrm{Pt_{C_\alpha}}@z_0$	9.62	−0.38	0.50
$\mathrm{Pt_{C_\beta}}@z_0$	9.36	−0.54	1.44
$\mathrm{Pt_{C_\beta C_\gamma}}@z_0$	9.23	−0.77	1.67
$\mathrm{Pt_T}@z_1$	10.11	0.11	−0.93
$\mathrm{Pt_{C_\alpha}}@z_1$	9.72	−0.28	1.43
$\mathrm{Pt_{C_\alpha C_\beta}}@z_1$	9.45	−0.55	0.81
$\mathrm{Pt_T}@z_2$	10.17	0.17	0.35
$\mathrm{Pt_{C_\beta}}@z_2$	9.59	−0.31	1.42
$\mathrm{Pt_{C_\beta C_\gamma}}@z_2$	9.56	−0.34	1.11
$\mathrm{Pt_{LB}}@a_1$	9.75	−0.25	0.44
$\mathrm{Pt_{(C_\alpha)^2}}@a_1$	9.76	−0.24	0.17
$\mathrm{Pt_{C_\alpha}}@a_2$	9.74	−0.26	1.33
$\mathrm{Pt_{(C_\alpha)^2}}@a_2$	9.78	−0.12	0.51

[1] G. Henkelman, A. Arnaldsson, and H. Jónsson, Computational Materials Science **36**, 354 (2006).

[2] E. Sanville, S.D. Kenny, R. Smith, and G. Henkelman, J. Comput. Chem. **28**, 899 (2007).

[3] W. Tang, E. Sanville, and G. Henkelman, J. Phys.: Condens. Matter **21**, 084204 (2009).

[4] M. Yu and D.R. Trinkle, J. Chem. Phys. **134**, 064111 (2011).
